# Preparation, characterization and utilization of coreshell super paramagnetic iron oxide nanoparticles for curcumin delivery

**DOI:** 10.1371/journal.pone.0200440

**Published:** 2018-07-18

**Authors:** C. Justin, Antony V. Samrot, Durga Sruthi P., Chamarthy Sai Sahithya, Karanam Sai Bhavya, C. Saipriya

**Affiliations:** Department of Biotechnology, Sathyabama Institute of Science and Technology, Jeppiaar Nagar, Rajiv Gandhi Salai, Chennai, Tamil Nadu, India; Universiti Putra Malaysia, MALAYSIA

## Abstract

In this study, super paramagnetic iron oxide nanoparticles (SPIONs) were produced by chemical co-precipitation method, then it was constructed to be a core shell nanoparticle by functionalizing with SDS, loading with curcumin and coating with a biopolymer i.e. chitosan. Each step was analyzed microscopically and spectroscopically. The produced coreshell particles were between 40 and 45nm and these coreshell particles were utilized for drug delivery studies against cervical cancer cell line—HeLa cells. The coreshell SPIONs were found to be releasing curcumin in between 6 and 12 h, which was evidenced by increased apoptotic cells and increased caspase 3 expression in HeLa cells.

## Introduction

Superparamagnetic iron oxide nanoparticles (SPIONs) are believed to be one of the promising candidates for various biomedical applications such as imaging, diagnosis, drug carrier for targeted drug delivery and many others [1–4]. Most of the drugs have the issue of reaching the target site, which is overcome by advanced drug delivery of modern medicine wherein, targeted drug delivery is possible when proper nanocarrier is used [5]. SPIONs are the potent candidates as nanocarriers for targeted drug delivery as they are safe, obey external magnetic field and also allow fabrication and surface engineering [5]. Even more, using SPIONs, deficiently bioavailable drugs can be loaded and directed to the diseased location or site of action of the drug with the support of an external magnetic field, thus cross the limitations attributed by conventional therapeutic tools [6,7]. Hence, SPIONs have been raised to be one of the most important nanoparticles in industrial and biomedical research.

One of the deficiently bioavailable drugs, despite its varied cytotoxic effect, is curcumin. This might be due to poor absorption, rapid metabolism and rapid systemic elimination of curcumin. The efficacy of curcumin for biological applications is studied in depth by many researchers [8–11]. Curcuminoids, which include curcumin related molecules, are effective antioxidants with demonstrated medicinal effects [12] and as anticancer agent against various cancer types [13,14]. These incredible versatile properties of curcumin made the researchers to use it in drug delivery applications. To make the drug bioavailable at the target site, it is required to be loaded on a proper carrier like SPIONs.

In order to load any drug onto SPIONs, it must be surface engineered or functionalized. This also increases its biomedical applications [15]. Surface engineering of particles enhance the drug loading and drug release efficiency and also reduce the non-specific or unpleasant interaction with host [2, 16]. Ionic, nonionic, cationic and amphoteric surfactants are well used for functionalization [17]. Sodium dodecyl sulfate (SDS) is one of the ionic surfactants which belongs to the amphiphiles family; they possess hydrophobic hydrocarbon chain and a polar head group [18].

In drug delivery applications of SPIONs, the coating agent protects nanoparticles from chemical reaction and also enhances its stability. More precisely, it prevents hydrophobic–hydrophobic interactions which leads to SPIONs aggregation [19,20]. Coating with biocompatible polymer makes it indispensable. Biopolymer like chitosan is a best choice, which is a natural, biodegradable, non-antigenic, non-toxic and bio-functional polymer [21]. However, the solubility of chitosan in acid solution limits the applications of this material [22]. Pyridoxine hydrochloride dissolves chitosan, moreover it is biocompatible. Pyridoxine, a water soluble vitamin involves in amino-acid, carbohydrate and fat metabolism and is also required for the formation of haemoglobin when given as hydrochloride [23]. Pyridoxine is usually given orally–the preferred route–and may also be given subcutaneous, intramuscular, or intra venous routes [24]. Owing to the ample biological usefulness of pyridoxine hydrochloride, in this study, chitosan was dissolved in it and was used for coating the curcumin loaded functionalized SPIONs as the final phase of the core shell preparation. The produced coreshell SPIONs were microscopically and spectroscopically characterized and determined for their IC50 value against HeLa cell line. The drug release ability and induction of apoptosis by core-shell were examined by apoptosis assay and caspase activity against HeLa cell line.

## Materials and methods

### Chemicals and reagents

For the synthesis of SPIONs, the following analytical grade and extra pure chemicals were used: Ferrous sulphate heptahydrate (FeSO_4_.7H_2_O), extra pure, from HiMedia laboratories, Ferric chloride hexahydrate (FeCl_3._ 6H_2_O) from Thomas Baker PVT limited; Tetramethyl ammonium hydroxide (C_4_H_13_NO), and Formaldehyde solution (CH_2_O) (41%), Acetone (C_3_H_6_O) from SDFCL,SDS (Sodium dodecyl Sulphate (C_12_H_25_NaO_4_S) from Karnataka Fine Chem, Bangalore, and N, N Dimethylformamide (HCON(CH_3_) from Fisher Scientific–Qualigens, Mumbai, Curcumin (C_21_H_20_O_6_) from SDFCL, Chitosan (C_6_H_11_NO_4_)_n_ (cell culture tested) from HiMedia, Pyridoxine hydrochloride (pure) (C_8_H_11_NO_3_HCL) and Curcumin from SRL (Sisco Research Laboratories) Mumbai, RPMI 1640 medium (#AL199A, HiMedia), Fetal Bovine Serum (RM10432), HiMedia), Caspase 3 (Cat No: 560901, BD Biosciences) and D-PBS (#TL1006, HiMedia). Throughout the synthesis of nanoparticles, nitrogenized double distilled water was used. For functionalization, drug loading and final polymer coating freshly prepared double distilled water was used. For giving proper agitation during incubation time a cyclo-rotator was designed to fit our purpose. For cell culture experiments, HeLa cell line was obtained from Stellixer Biotec, Bengaluru, India.

### Synthesis of core-shell nanoparticles

#### Synthesis of SPIONs

SPIONs were prepared in the magnetic field having precursor molecular solution of 0.0132% as per our earlier report [25].

#### Stabilization and functionalization of SPIONs

0.080 g SDS powder was dissolved in 100 mL water, vortexed and kept undisturbed until the foam turned into solution. It was warmed in water bath at 70°C for 20 minutes and then centrifuged at 10000 g, the obtained clear solution was collected and used further. Meanwhile, 0.008 g of magnetically separated SPIONs weres washed four times with double distilled water and washed with 5 mL N, N dimethylformamide. The SPIONs were separated with magnets and dispersed into the freshly prepared 20 mL SDS and gently mixed for five minutes, followed with mixing in cyclo-rotator for every 10 minutes/hour for three days.

#### Curcumin loading

0.020 g curcumin powder was dissolved in 2 mL of N, N Dimethylformamide. 400 μL of curcumin from above solution was decanted into the SPIONs-surfactant (SDS) mixture and mixed gently for five minutes and kept in the cyclo-rotator for three days. After three days, the resultant curcumin loaded SPIONs were transferred into 2 mL microcentrifuge tubes and warmed in water bath at 70°C for 20 minutes. At every three minutes interval, the mixture was gently shaken to permeate the temperature uniformly. Curcumin loaded particles were magnetically separated and washed thrice with 0.1% pyridoxine hydrochloride and dispersed into 5 mL of 0.1% pyridoxine hydrochloride solution.

#### Coating with biopolymer

Chitosan (0.67 g) was dissolved into 20 mL of 1.67% pyridoxine hydrochloride solution. The obtained gelatinous polymer was centrifuged at 3000 g for 10 minutes and the resultant supernatant was used for coating. Curcumin loaded SPIONs was decanted into 20 mL of above gelatinous biopolymer and gently mixed for five minutes and kept in the cyclo-rotator for three days. After incubation, the particles were magnetically separated and dispersed into 5 mL of 0.1% pyridoxine hydrochloride solution.

### Analytical methods

UV Vis absorbance for the samples were analyzed between 200 and 800 nm in Shimadzu UV Vis NIR Spectrophotometer. The samples were loaded onto a clean glass slide, FTIR spectra was recorded as transmission mode scan for the spectral region between 4000 and 500 cm^-1^ using IR Affinity 1S (Shimadzu, Japan). Raman spectra was recorded having 785 nm diode laser using LabRam HR 800 model (Horiba Jobinyvon). Sample was loaded onto a clean glass slide and XRD spectra was recorded using Smart lab XRD (Rigaku). To study the stability of the produced nanoparticle, Zeta potential analysis was done in Brookhaven ZetaPALS. Prior to imaging, all the samples were dried on aluminum foil and coated with gold using Quorum, Q 150R ES. SEM images of all the samples were taken in Zeiss Ultra55. All the synthesized samples were loaded onto a clean glass coverslip and AFM images was recorded using Bruker, Dimension icon model.Magnetic measurements of the synthesized samples at field cooled (FC) and zero field cooled (ZFC) was recorded.XPS analysis for Fe, carbon and oxygen–spectrum of synthesized nanoparticles was performed using Ultra DLD model of Kratos.

### In vitro drug delivery studies

#### Cell line used

HeLa cell line (Stellixer Biotech, Bangalore) was used to study the viability, apoptotic, and gene expression induced by the test compound (Curcumin encapsulated core-shell).

#### MTT assay

100 μL of cell suspension was seeded into a 96 well plate at required cell density (2x10^4^ cells/well) and allowed to grow for 12 h. Different concentration of core-shell was added to the cell culture and the total volume in a well was made into 200 μL using media. The plate was then incubated at 37°C for 24 h in a 5% CO_2_ incubator. After incubation, the media was pipetted out, 10 μL of 10% MTT was added and incubated for two hours in a dark place. After 2 h, MTT reagent was removed and added with 100 μL of solubilizing agent (DMSO). The absorbance was recorded in an ELISA reader at 570 nm and 630 nm was used as reference wavelength [26]. The IC50 value was determined from the cell viability graph.

#### Apoptotic assay

HeLa cells were cultured in a 6 well plate at a density of 3x10^5^ cells/2 ml and incubated in CO_2_ incubator at 37°C for 24h. After removing the spent media, the concentration of core-shells obtained through IC50 value was added to the well and incubated for analyzing the apoptotic effect for four time intervals (2, 6, 12, 24h). After incubation, the cells were trypsinised and the cell pellet was washed with PBS and added with Annexin V and Propidium iodide (Apoptosis detection kit I, 556547, BD Biosciences) in the dark as recommended by the manufacturer [27,28]. The cells were then analysed using flow cytometer (BD FACS Caliber) for their viability, early apoptotic and late apoptotic states.

#### Gene expression (caspase 3) assay

The cells were cultured in RPMI medium in a 6 well plate at a density of 3 x 10^5^ cells/2 mL and incubated in a CO_2_ incubator at 37°C for 24h. The spent medium was then aspirated and the cells were treated with the core-shells dispersed in fresh medium and retrieved for caspase-3 expression accordingly for four time intervals (2, 6, 12, 24h). At each time interval, the medium was removed from the wells, washed with PBS and trypsinised. Harvested cells were centrifuged at 2000 rpm and supernatant was decanted. Caspase expression was estimated using FITC Rabbit Anti- Active Caspase– 3 (BD Biosciences, Catalog no.560901). Further analysis was done in flow cytometry (BD FACS Caliber).

## Results and discussion

### UV visible spectroscopy

**Fig 1:**
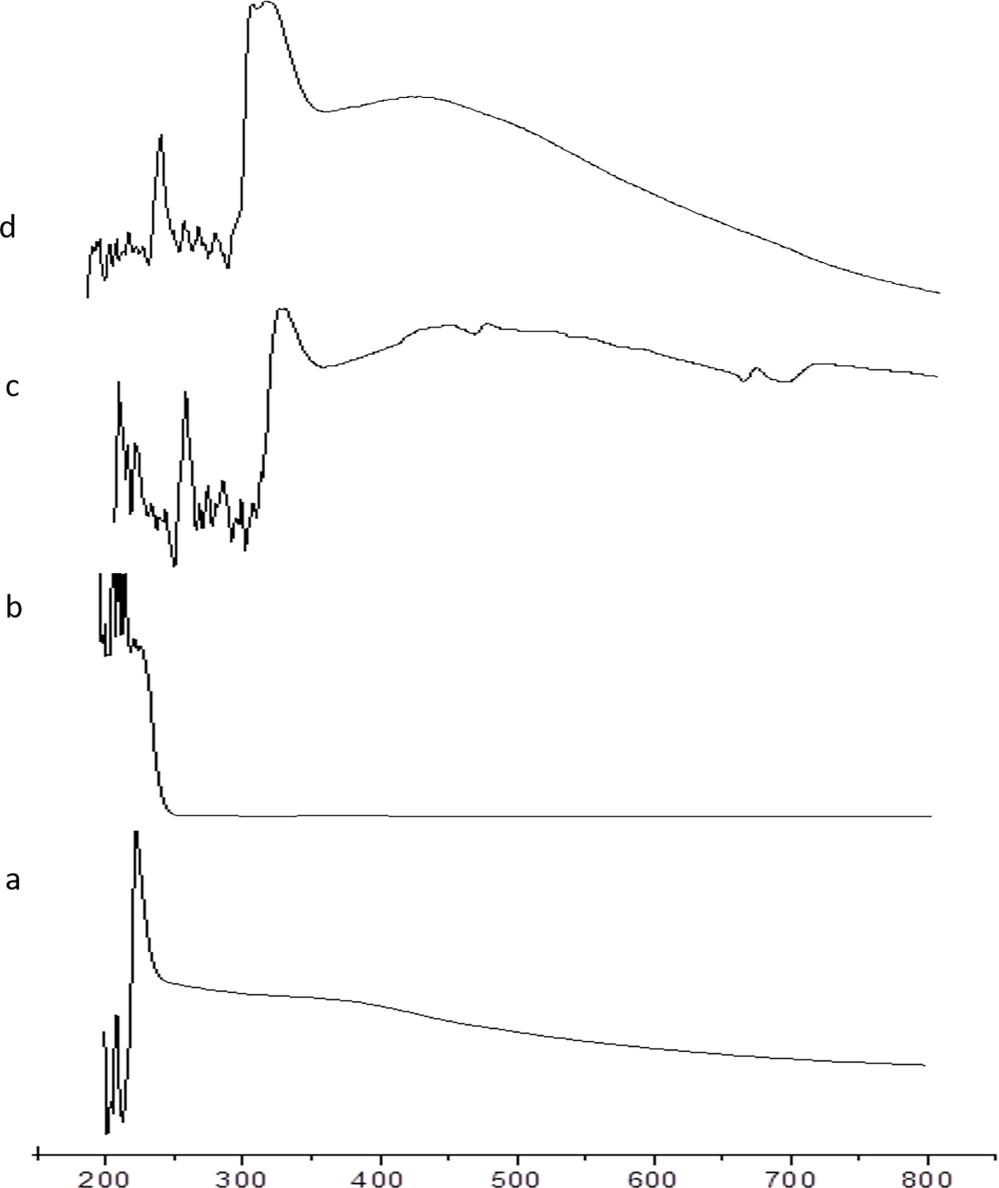
UV vis spectra of the four phases of coreshell preparation: (a) SPIONs, (b) after functionalizing with SDS, (c) after curcumin loading, (d) after encapsulating with biopolymer coating.

The absorption maximum of SPIONs was found to be 250 nm (Fig 1A) and this absorption was completely concealed by SDS (Fig 1B). This stated that the surfactant was completely masking the SPIONs and funtionalized the SPIONs (Fig 1B). The drug used here was curcumin and its absorbance was observed between 460 and 440 nm (Fig 1C), which was the spectral signature of curcumin loaded on to the funtionalized SPIONs [29]. After the final coating with biopolymer, there was a sharp peak arround 270nm supporting the presence of chitosan [30] and the peak at 430nm along with the shoulder peak towards 450nm supported the gelatinous nature of chitosan & pyridoxine hydrochloride compound (Fig 1D).

### FTIR

**Fig 2:**
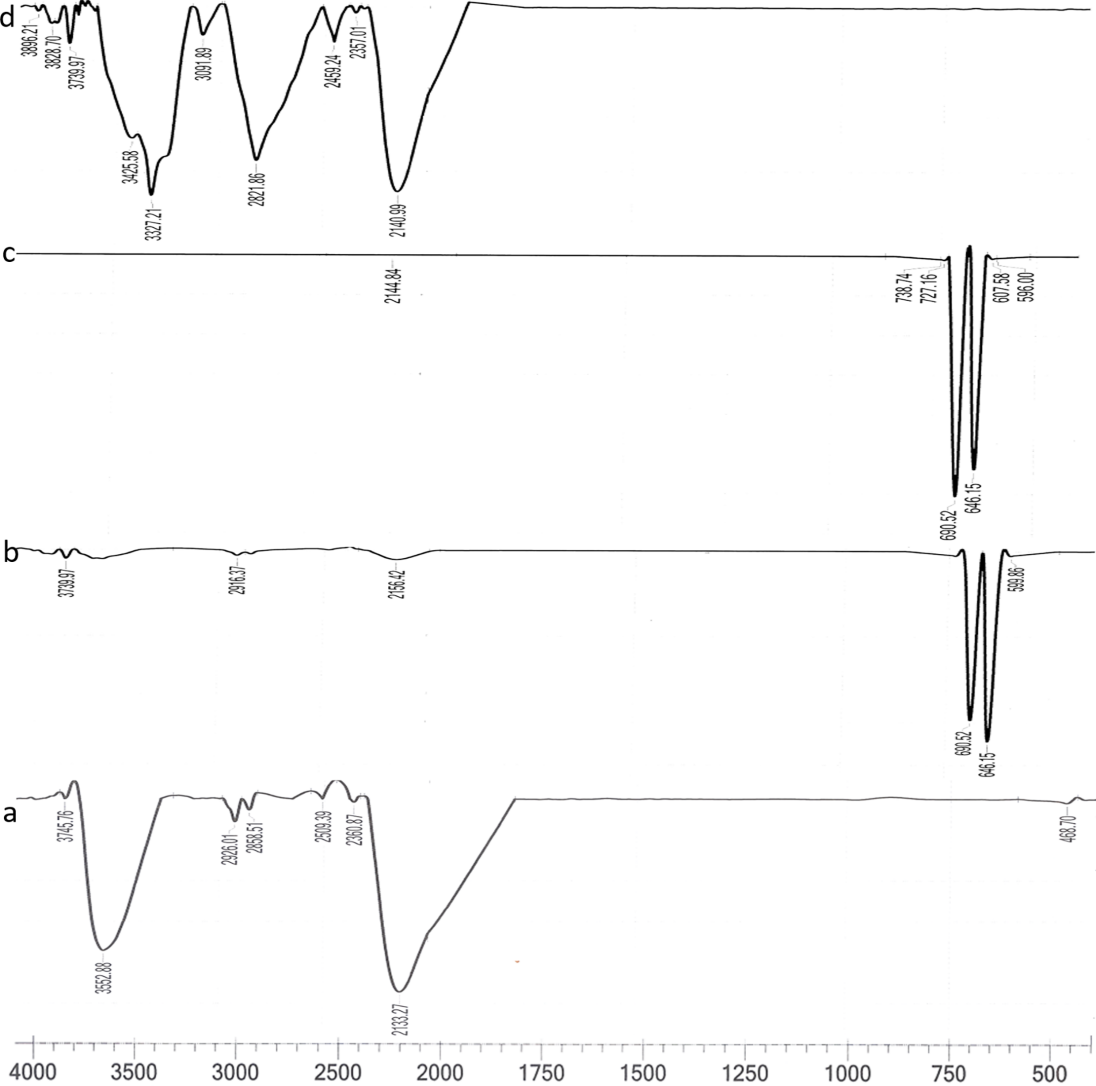
FTIR spectra of the four phases of coreshell preparation: (a) naked SPIONs, (b) after functionalizing with SDS, (c) after curcumin loading, and (d) after encapsulating with biopolymer coating.

The peak at 3745.76 and 2133.27 cm^-1^ was considered as the finger print region of the iron oxide nanoparticles (Fig 2A). Most bands were for O-H stretching (3552.88 cm^-1^) and -C≡C- which required for bond formation at ≡Fe-OH sites [25,31]. The emergence of new finger print region at 500 to 750 cm^-1^ while maintaining some of the absorption regions of the naked SPIONs supports the association of both the anchoring and the charging groups which involved in functionalization by SDS (Fig 2B), when any organic molecules are used to functionalize SPIONs, they usually have two feature groups i.e. the anchoring group and the charging group. The former anchors itself onto the nanoparticles’ surface, while charging group binds to the respective charges dependent on the charge density, modified structure, and chain length [32]. The IR spectra for -CH of aromatic ring of curcumin was found near 738 cm^-1^ and 727 cm^-1^. After chitosan coating, the finger print region was shifted to IR spectral range, i.e., 2000 to 4000 cm^-1^ which was the characteristic spectra of the biopolymer used in this experiment (Fig 2D).

### Raman spectroscopy

**Fig 3:**
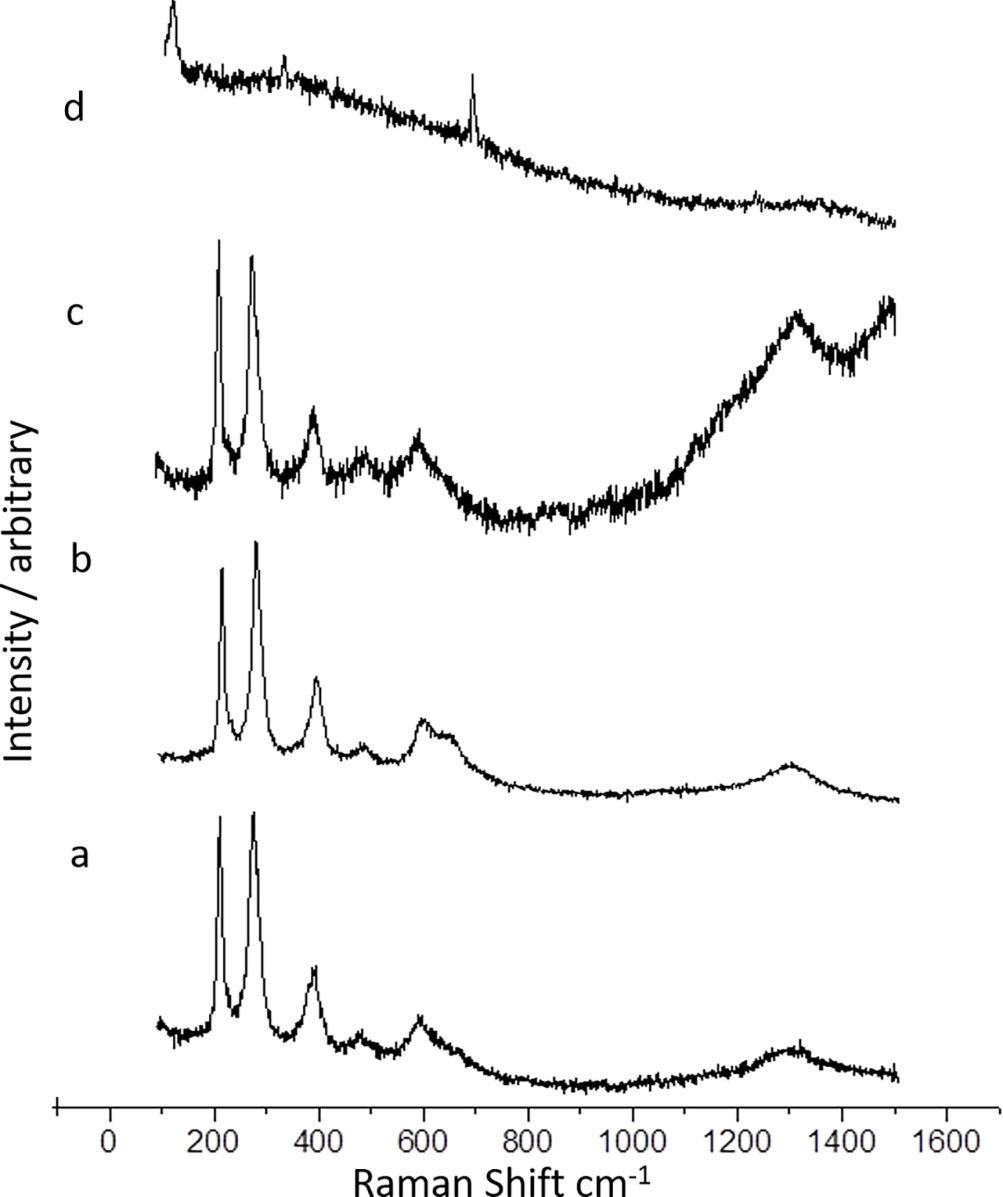
Raman spectra of the four phases of coreshell preparation: (a) naked SPIONs, (b) after functionalizing with SDS, (c) after curcumin loading, (d) after encapsulating with biopolymer coating.

Peaks at 213, 274, 384 and 474 cm^-1^in Eg mode and peak at 584 cm^-1^in A1g mode represented iron oxide (Fig 3A), where these peak ranges were already reported [25,33]. In Fig 3B, in addition to the respective peaks for iron oxide, there observed a shoulder peak at 635 cm^-1^ which was due to SDS of functionalized SPIONs. After loading of curcumin, a steady upturn after 1100 to 1300 cm^-1^ and another down turn and upturn in between 1300 and 1500 cm^-1^ were observed (Fig 3C). This could be validated with the Raman spectrum taken for the dissolved curcumin. In Fig 3D, the Raman spectra taken after final coating with biopolymer exhibited two partial peaks at 500 and 300 cm^-1^ and absolutely masked the whole SPIONs and curcumin, which was indication of formation of core-shell.

### XRD

**Fig 4:**
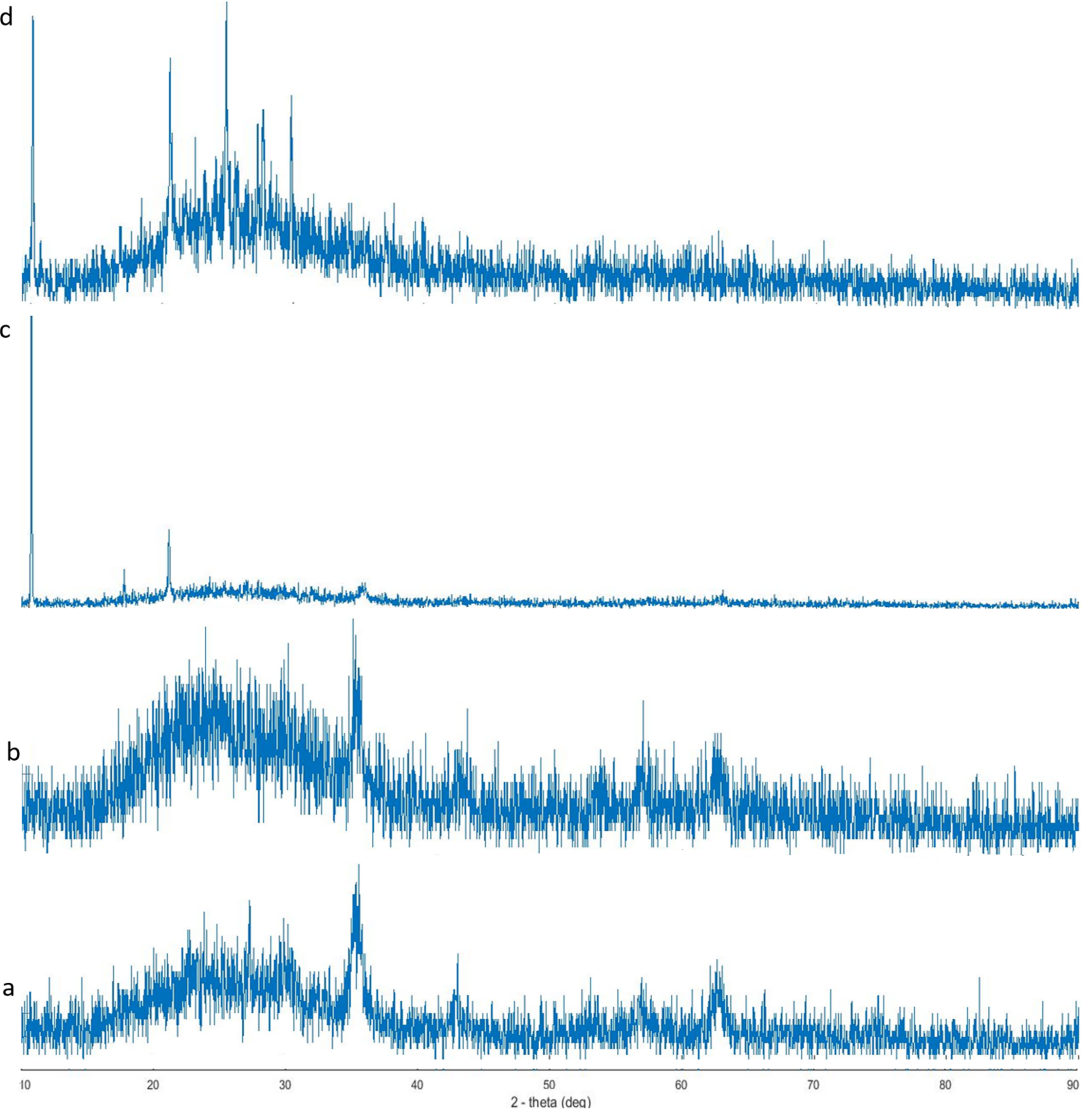
XRD spectra of the four phases of coreshell preparation: (a) naked SPIONs, (b) after functionalizing with SDS, (c) after curcumin loading, (d) after encapsulating with biopolymer coating.

The state of crystallinity was investigated all through the four phases of the core-shell preparation. As reported earlier, the produced SPIONs was γ-Fe2O3 [25,34]. In Fig 4B, peaks were relevant for the crystallinity of the SPIONs as even they were shielded with SDS molecule but the peaks were slightly nebulous than Fig 4A. The crystalline nature was preserved even after curcumin loading on to the functionalized SPIONs (Fig 4C) and after the final coating with biopolymer (Fig 4D).

### ZETAPOTENTIAL

Nanoparticles with zeta potential values greater than +25 mV or less than –25 mV show high degrees of stability, where the particles with low zeta potential value aggregate due to Van der Waal inter particle attractions [35]. The zeta potential value of the naked SPIONs was –15 mV (S1 Fig), while functionalizing with SDS, the zeta potential value also was observed increasing (S1 Fig). Similar phenomenon has been already reported when the surfactants were used to disperse nanoparticles entirely in an appropriate medium [31]. Coating single domain of iron oxide nanoparticles, with double layer of surfactants in an aqueous medium result in stable colloidal dispersions. After loading curcumin, further increase in stabilization along with charge-reversal was observed (S1 Fig). Such kind of increase in stabilization while drug loading is already reported [36]. Moreover, after loading curcumin, the surface charge-reversal from anionic to cationic was due to the cationic nature of curcumin. Even the encapsulation of curcumin loaded SPIONs with the biopolymer (pyridoxine hydrochloride & chitosan) increased the stabilization and the charge remained cationic (S1 Fig). Moreover, it is suggested that Zeta-potential analysis of surface potentials over ±20 mV is sufficient to be repulsive in neutral solvent [37].

### SQUID

Superparamagnetic state was maintained through all phases of core-shell preparation. The shape of the hysteresis curve for the four phases, i.e., for naked SPIONs (S2 Fig), after functionalization (S2 Fig), after curcumin loading (S2 Fig), and after the polymer coating (S2 Fig) showed its magnetization was lesser than larger particles as reported [38,39]. Temperature dependent magnetization (FC and ZFC) for the four phases of core-shell preparation was also studied. In field cooled magnetization, a slight fluctuation in magnetization was observed for the naked SPIONs (S3 Fig). Over the other three phases saturation magnetization was observed around 250K-260K and started lowering after that (S3 Fig). Coating the magnetite nanoparticles with either metal or polymers reduces magnetization values [40–42]. In zero field cooled magnetization, a common saturation magnetization was found at 350K (S4 Fig), these SPIONs can be used for drug delivery as well as hyperthermia mediated cell death, as they are withstanding 315K [43,44].

### XPS

The chemical states of Fe, over the phases of core-shell preparation is presented in S5 Fig. The binding energy of Fe2p_3/2_ /eV was found at 706.7, 709.6, 710.8, and 710.4 eV, which were for Fe, FeO, Fe_2_O_3_, and FeCl_2_ respectively. According to the spectra, the higher binding energy closer to FeCl_2_ was Fe_2_O_3_. As per the four spectra the original chemical state of SPIONs was preserved until the completion of the core-shell preparation. Oxygen atoms are part and parcel of iron oxide compounds. XPS spectra taken for oxygen of the four phases of core-shell preparation is given in S5 Fig. Particularly for naked SPIONs the binding energy for oxygen occurs from 529 to ~ 535 eV (S6 Fig) creating the broad spectrum. The binding energy in between 529–530 eV was because of metal oxide, whereas the other energy states at 531.5 to 532 eV was for C-O, ~ 533eV was for C = O and ~ 535 was for O-F_x_. After functionalization with SDS, the spectrum for oxygen resembles very close to that of the oxygen’s energy state in the naked SPIONs (S6 Fig). After curcumin loading there was a considerable change in the spectrum for oxygen in between 529 to ~ 535 eV (S6 Fig) which was due to the accumulation of oxygen from curcumin (C_21_H_20_O_6_). The steady appearance of the XPS spectrum derived in between 529 to ~ 535 eV, which was taken after the final coating with biopolymer (S6 Fig) substantiated the regularity and consistency of oxygen atoms in the core-shell level.

During chemical synthesis of SPIONs adventitious carbon contamination were likely to occur (S7 Fig). There were three chemical states at 284.8, ~ 286 and ~ 288.5 for C = C, C-O-C and O-C = O respectively which were observed. After functionalization with SDS, the spectrum for carbon atoms resembles very close to that of the carbon’s energy state in the naked SPIONs (S7 Fig). After curcumin loading, there was a considerable change in the spectrum for carbon (S7 Fig) though its binding energy state was maintained. This is probably due to the accumulation of carbon from curcumin molecules. The spectrum taken for carbon after biopolymer coating (S7 Fig) shows the addition of carbon atoms preserving the same energy state respectively for C = **C**, C-O-C, O-C = O.

### Scanning electron microscopy- EDX

**Fig 5:**
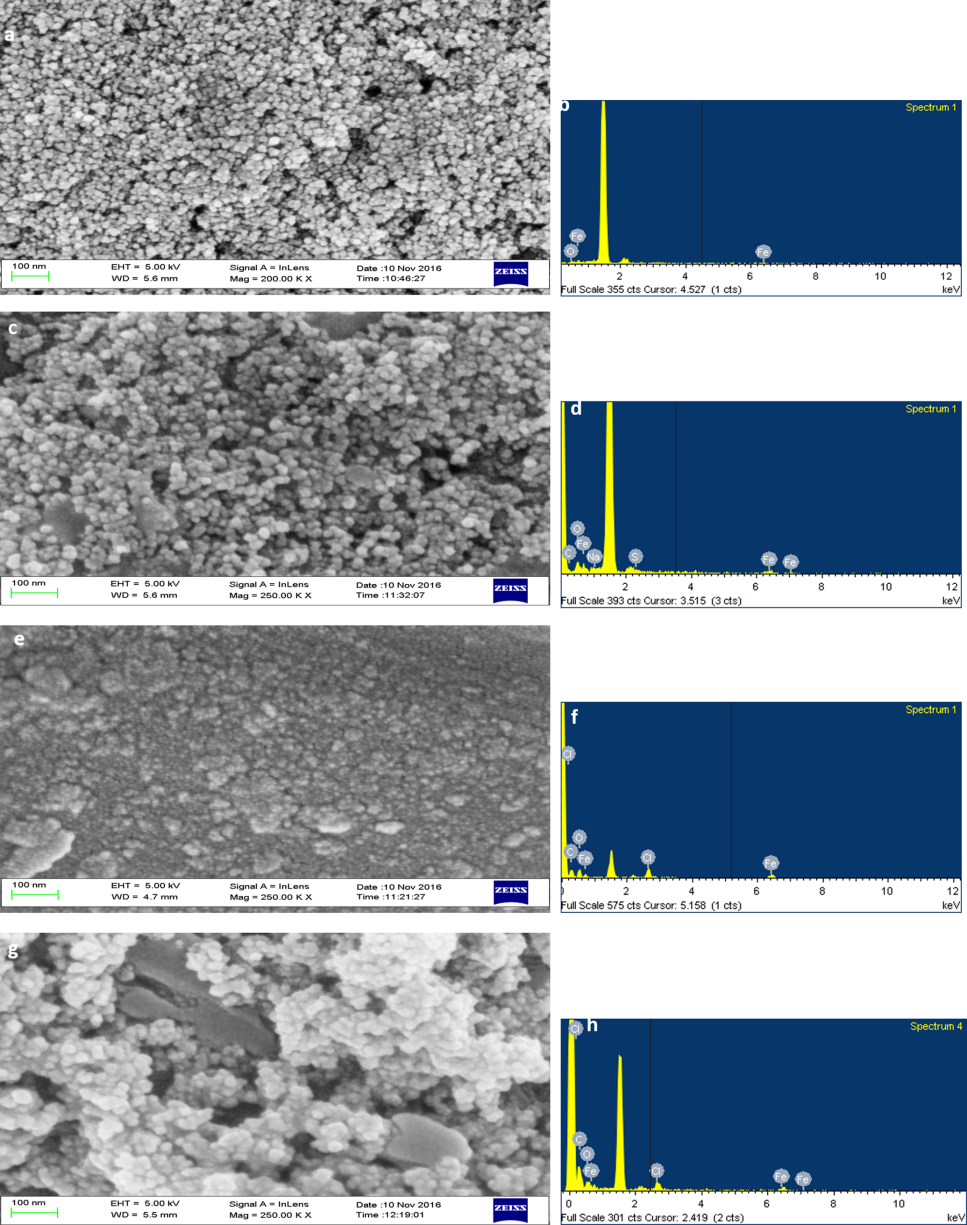
(a) SPIONs, (b) EDX spectrum of SPIONS, (c) SPIONs functionalized with SDS, (d) EDX spectrum of SPIONs functionalized with SDS (f) EDX spectrum of SPIONs functionalized and loaded with curcumin (g) SPIONs functionalized, loaded with curcumin and encapsulated with biopolymer coating, (h) EDX spectrum of SPIONs functionalized, loaded with curcumin and encapsulated with biopolymer coating.

SPION was found to be size between 10 and 15nm (Fig 5A). Functionalization has increased the size upto 20nm. In a study, Fe_3_O_4_ was found to be size of 7nm, when Fe_3_O_4_ was added with SiO_2_ and (3-Aminopropyl) triethoxysilane it became 40nm sized [45], which states that functionalization increased the size of the particles. When the drug loading was done, it increased the size to 35nm. At last the coreshell was made by coating with chitosan and the size was between 40 and 45nm (Fig 5G). Size below 100nm is always considered to be good for drug delivery.

### AFM

**Fig 6:**
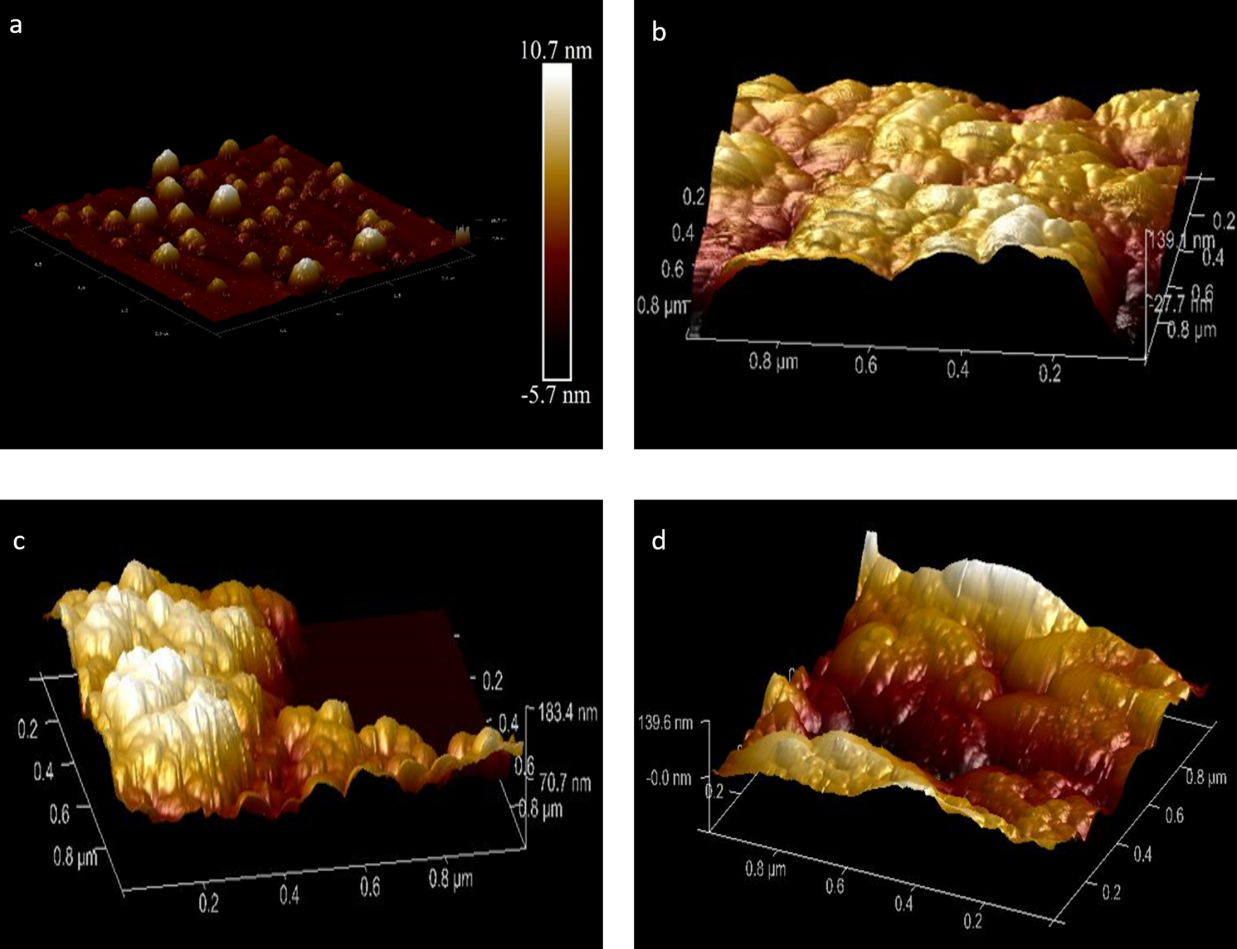
AFM images of the four phases of coreshell preparation. (a) SPIONs, (b) after functionalizing with SDS, (c) after curcumin loading, (d) after encapsulating with polymer coating.

AFM image was on par with the SEM result but showed bit aggregation of particles (Fig 6).

### In vitro drug delivery studies

#### MTT assay

**Fig 7:**
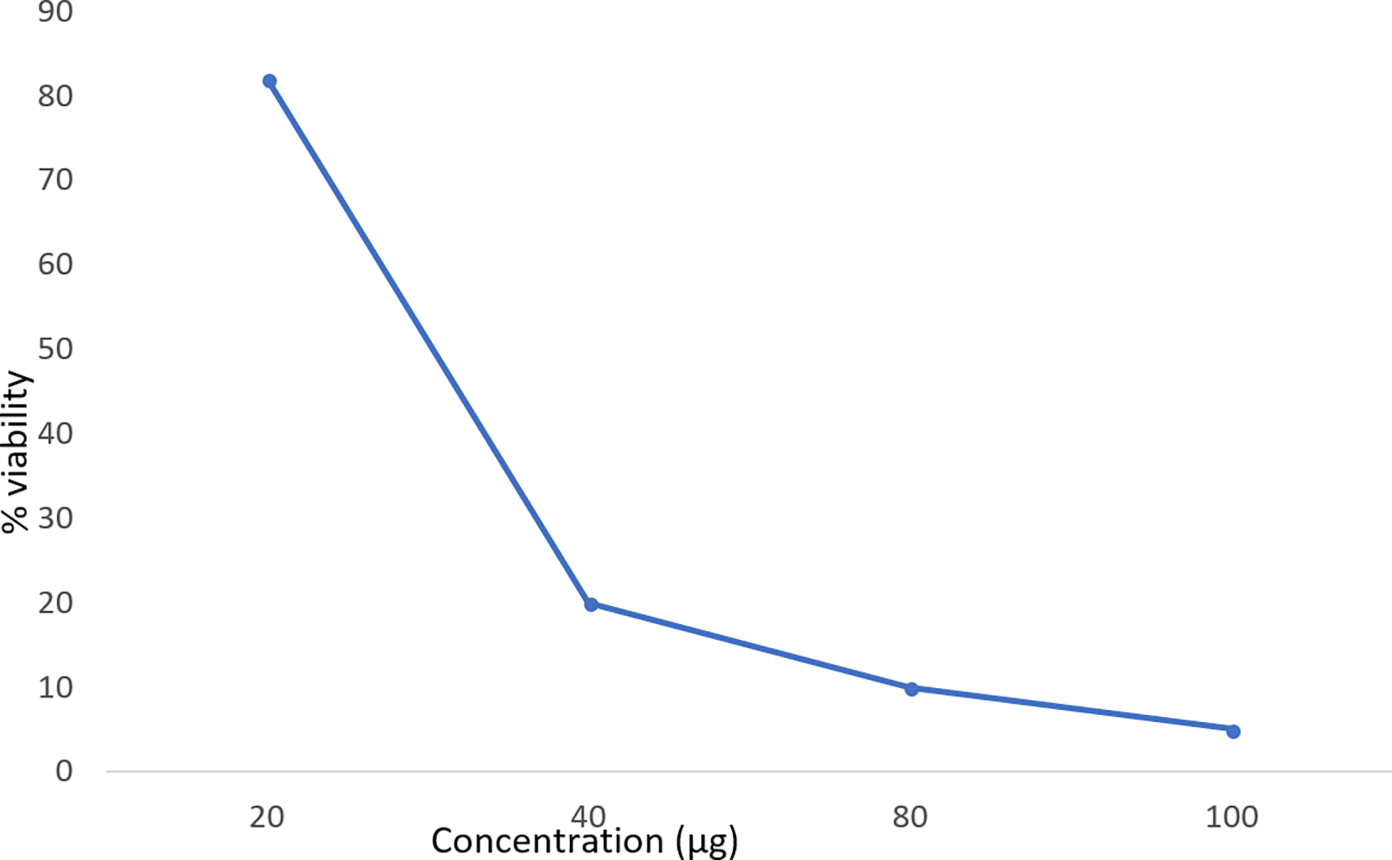
MTT assay done for the curcumin (drug) encapsulated coreshell in HeLa cell line.

As the concentration of the coreshell increased, the cell viability was found to be decreased (Fig 7), this might be because of release of loaded drug. Curcumin has been reported to induce DNA damage mediated cell death in HeLa cells in vitro [46]. The IC50 (inhibitory concentration) value of curcumin encapsulated core-shell was found to be 30μg.

#### Apoptotic assay

**Fig 8:**
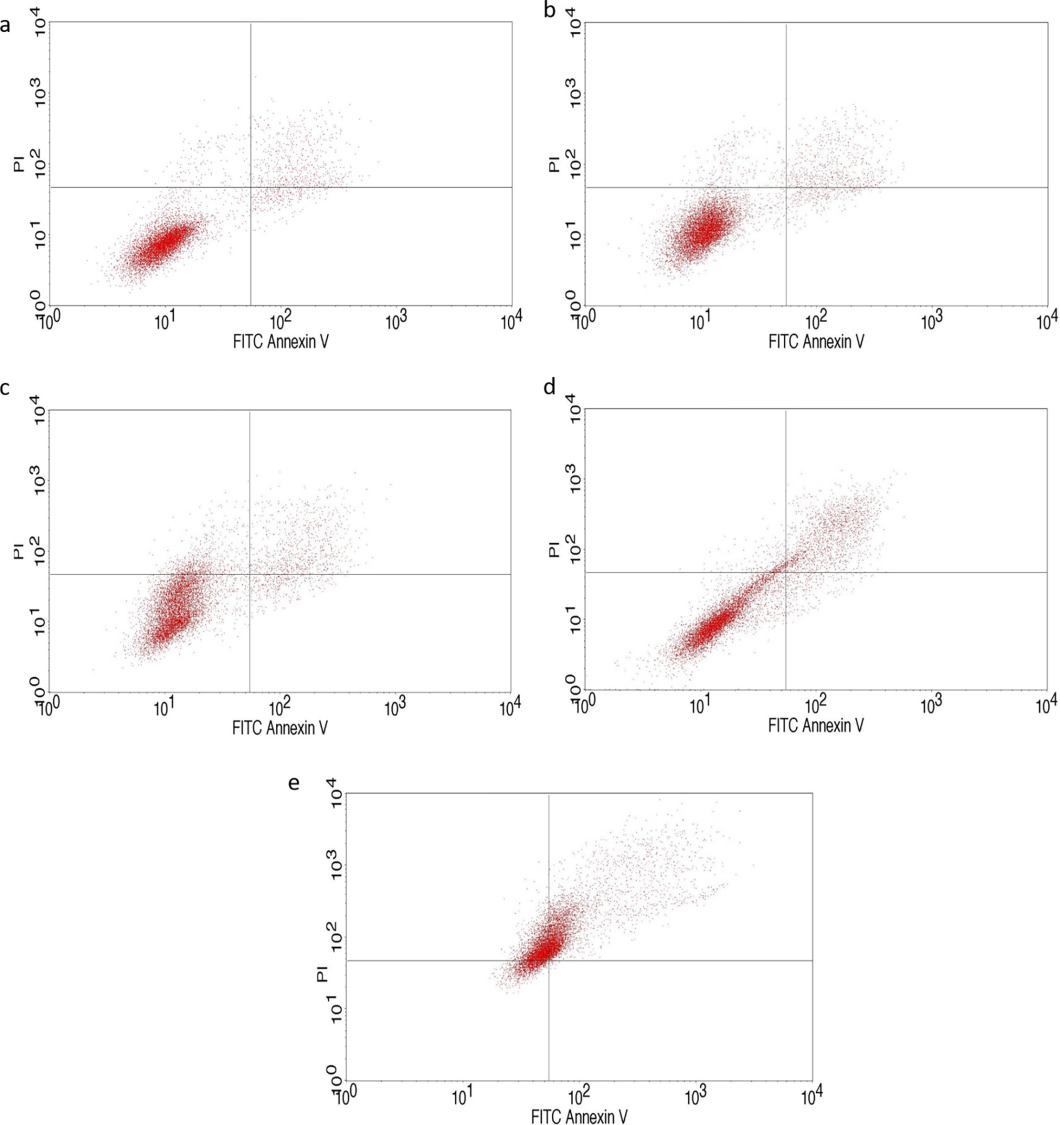
Flow cytometric analysis of the time dependent apoptotic assay in HeLa cell line: (a) control–not treated (b) HeLa cell treated with coreshell for 2 hours, (c) HeLa cell treated with coreshell for 6 hours, (d) HeLa cell treated with coreshell for 12 hours, (e) HeLa cell treated with coreshell for 24 hours.

IC50 concentration obtained through MTT assay was used for performing apoptotic assay. Accordingly, the state of the respective HeLa cell population is given in Fig 8. Untreated cells incubated for 24 hours were taken as control (Fig 8A). In between 2 and 6 hours there was only a slight difference observed. After 12 hours incubation, there was increase of apoptotic cells to above 15% and it was increasing to above 44% in 24h. Increase in the duration of the drug exposure decreased the viability of the cells, which implies the release of the drug into the medium as the time increases and had impact on the cells. Rajan et al [47] have also found camptothecin loaded magnetite nanoparticle to induce apoptosis against HeLa cells and they found magnetite to be a better drug delivering agent.

#### Caspase 3 expression assay

**Fig 9:**
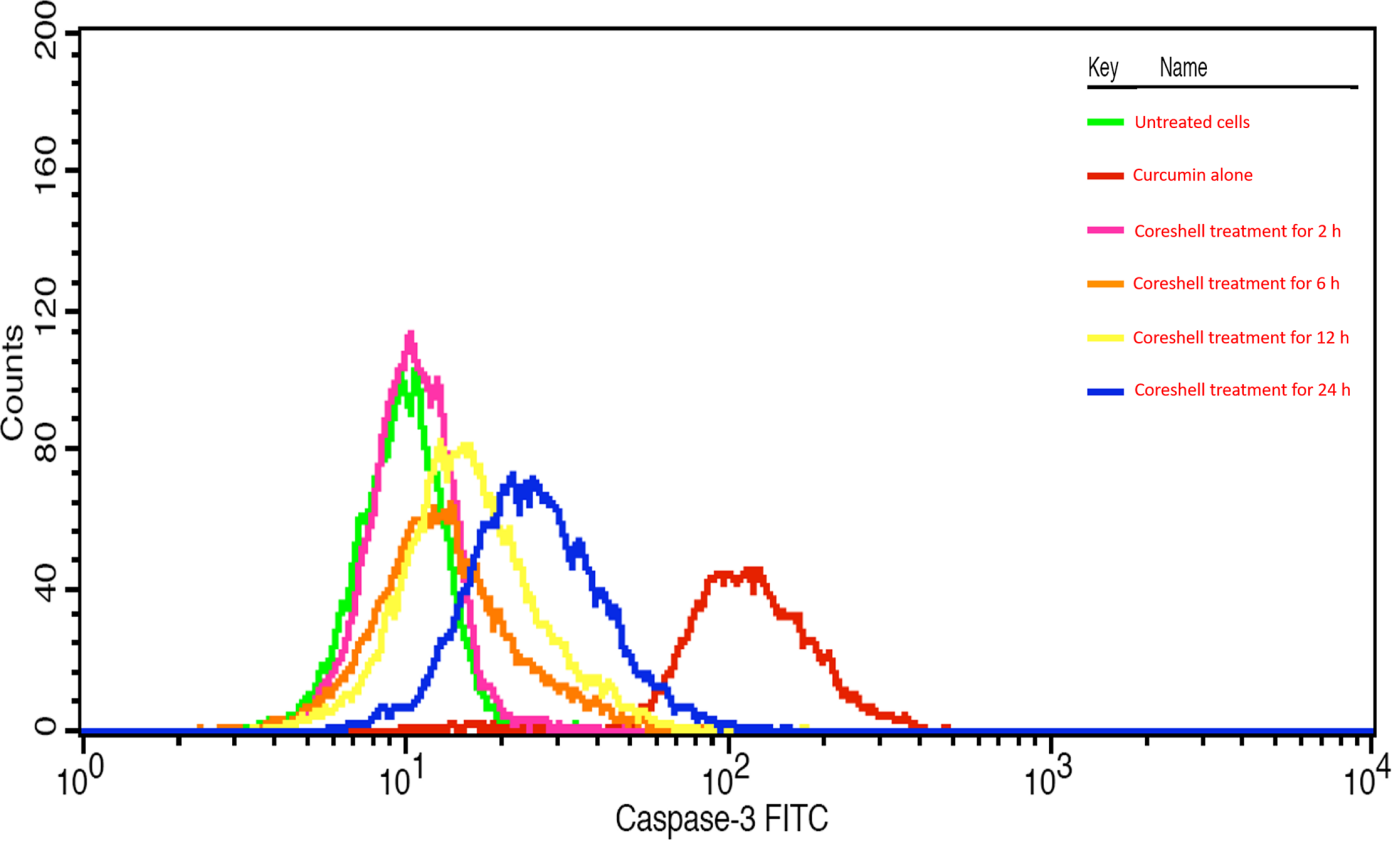
Caspase 3 expression assay of time dependent drug release in HeLa cell line.

There was no effect shown by coreshell in 2 h. There was steady increase in the expression of caspase 3, which was supporting the apoptotic assay (Fig 9). Rajan et al [47] have also found camptothecin loaded magnetite nanoparticle to increase the caspase expression to double fold in treated HeLa cells.

## Conclusion

Curcumin loaded core-shell SPIONs were synthesized and analyzed spectroscopically and microscopically. Morphological changes were observed in all the important phases of core-shell preparation. Required strength and crystallinity was conserved throughout all the phases. The surface charge was found to be cationic and the superparamagnetic property was maintained throughout. IC 50 value of the curcumin loaded core-shell against HeLa cells was determined as 30μg/mL. The ability of drug delivery by coreshell was determined by apoptotic assay and caspase 3 expression. The coreshell was able to deliver curcumin after 6h of incubation, which was evidenced by increased apoptotic cells and Caspase 3 expression. Using these coreshell SPIONs treating cancer cells at its site is possible, as this coreshell can be guided to the target site by external magnetic field and the bioavailability of curcumin at the target site is more.

## Supporting information

S1 Fig Zeta potential analysis of the coreshell preparation. (a) SPIONs after functionalizing with SDS, (b) after curcumin loading, (c) after encapsulating with biopolymer coating.
(TIF)

S2 Fig Magnetic hysteresis obtained from SQUID: (a) naked SPIONs, (b) after functionalizing with SDS, (c) after curcumin loading, (d) after encapsulating with biopolymer coating.
(TIF)

S3 Fig Magnetization measurement of SPIONs (ZFC mode) through the coreshell preparation: (a) naked SPIONs, (b) after functionalizing with SDS, (c) after curcumin loading, (d) after encapsulating with biopolymer coating.
(TIF)

S4 Fig Magnetization measurement of SPIONs (FC mode) through the coreshell preparation: (a) naked SPIONs, (b) after functionalizing with SDS, (c) after curcumin loading, (d) after encapsulating with biopolymer coating.
(TIF)

S5 Fig XPS spectra for the chemical states of Fe over the phases of coreshell preparation: (a) SPIONs, (b) after functionalizing with SDS, (c) after loading curcumin, (d) after encapsulating with biopolymer coating.
(TIF)

S6 Fig XPS spectra for the chemical state of oxygen over the coreshell preparation: (a) SPIONs, (b) after functionalizing with SDS, (c) after loading curcumin, (d) after encapsulating with biopolymer coating.
(TIF)

S7 Fig XPS spectra for the chemical state of carbon over the coreshell preparation: (a) SPIONs, (b) after functionalizing with SDS, (c) after loading curcumin, (d) after encapsulating with biopolymer coating.
(TIF)
