# Supplementary figures and images for: Preparation, characterization and utilization of coreshell super paramagnetic iron oxide nanoparticles for curcumin delivery
Source: PLoS One. 2018 Jul 18;13(7):e0200440. doi: 10.1371/journal.pone.0200440 (PMC6051608; doi:10.1371/journal.pone.0200440)

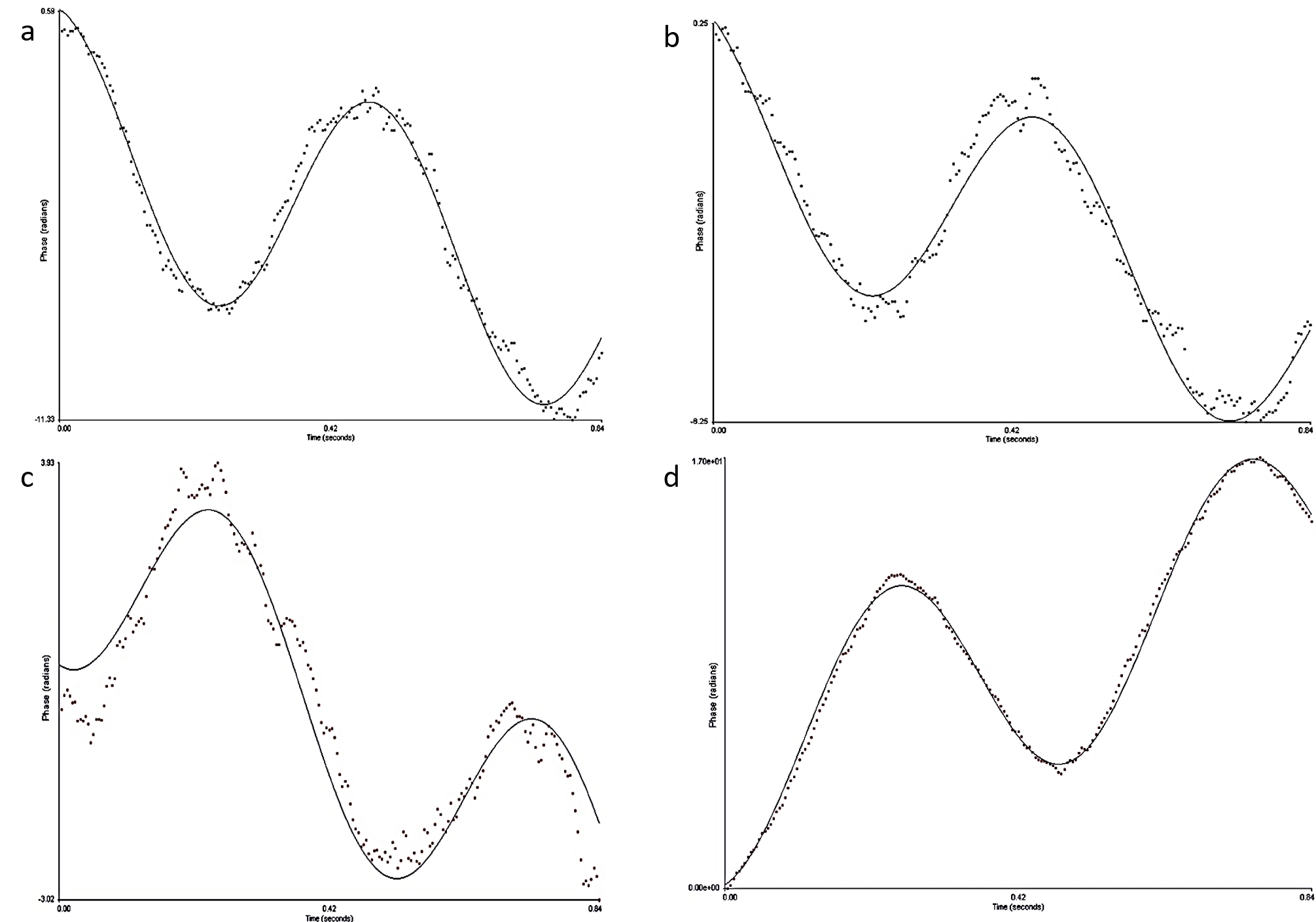

Supplement: S1 Fig — (a) SPIONs after functionalizing with SDS, (b) after curcumin loading, (c) after encapsulating with biopolymer coating. (TIF) [file pone.0200440.s001.tif]

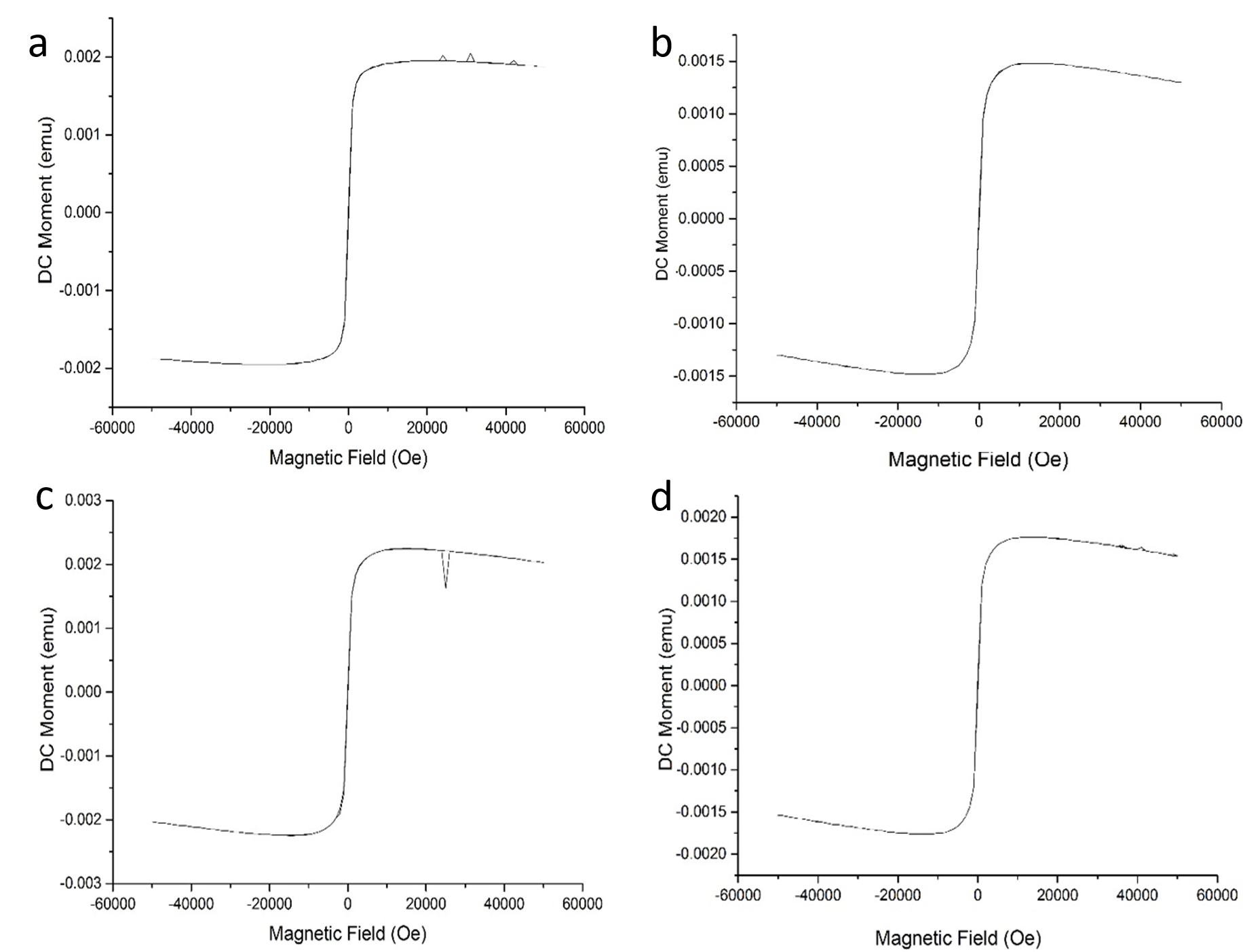

Supplement: S2 Fig — Magnetic hysteresis obtained from SQUID: (a) naked SPIONs, (b) after functionalizing with SDS, (c) after curcumin loading, (d) after encapsulating with biopolymer coating. (TIF) [file pone.0200440.s002.tif]

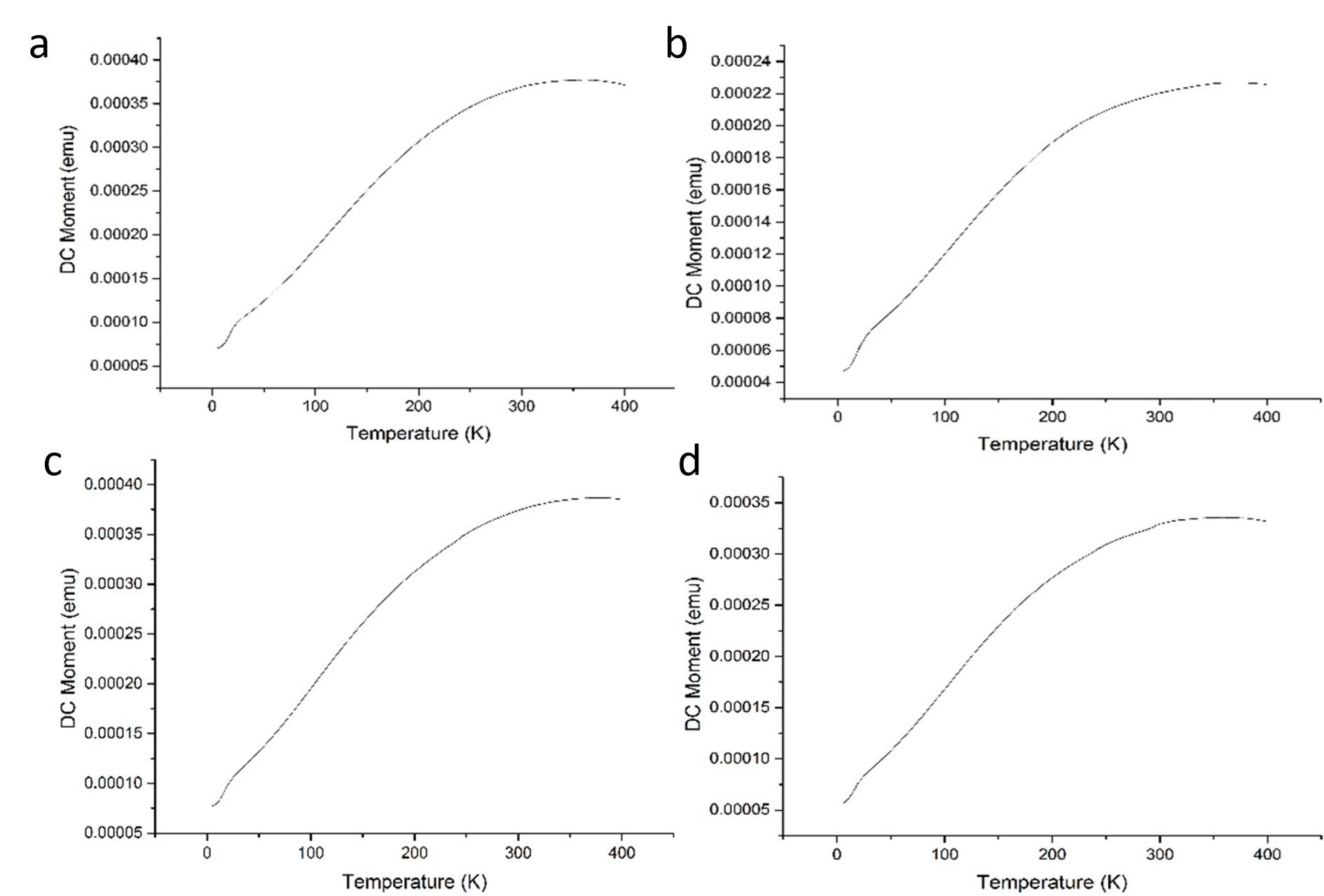

Supplement: S3 Fig — Magnetization measurement of SPIONs (ZFC mode) through the coreshell preparation: (a) naked SPIONs, (b) after functionalizing with SDS, (c) after curcumin loading, (d) after encapsulating with biopolymer coating. (TIF) [file pone.0200440.s003.tif]

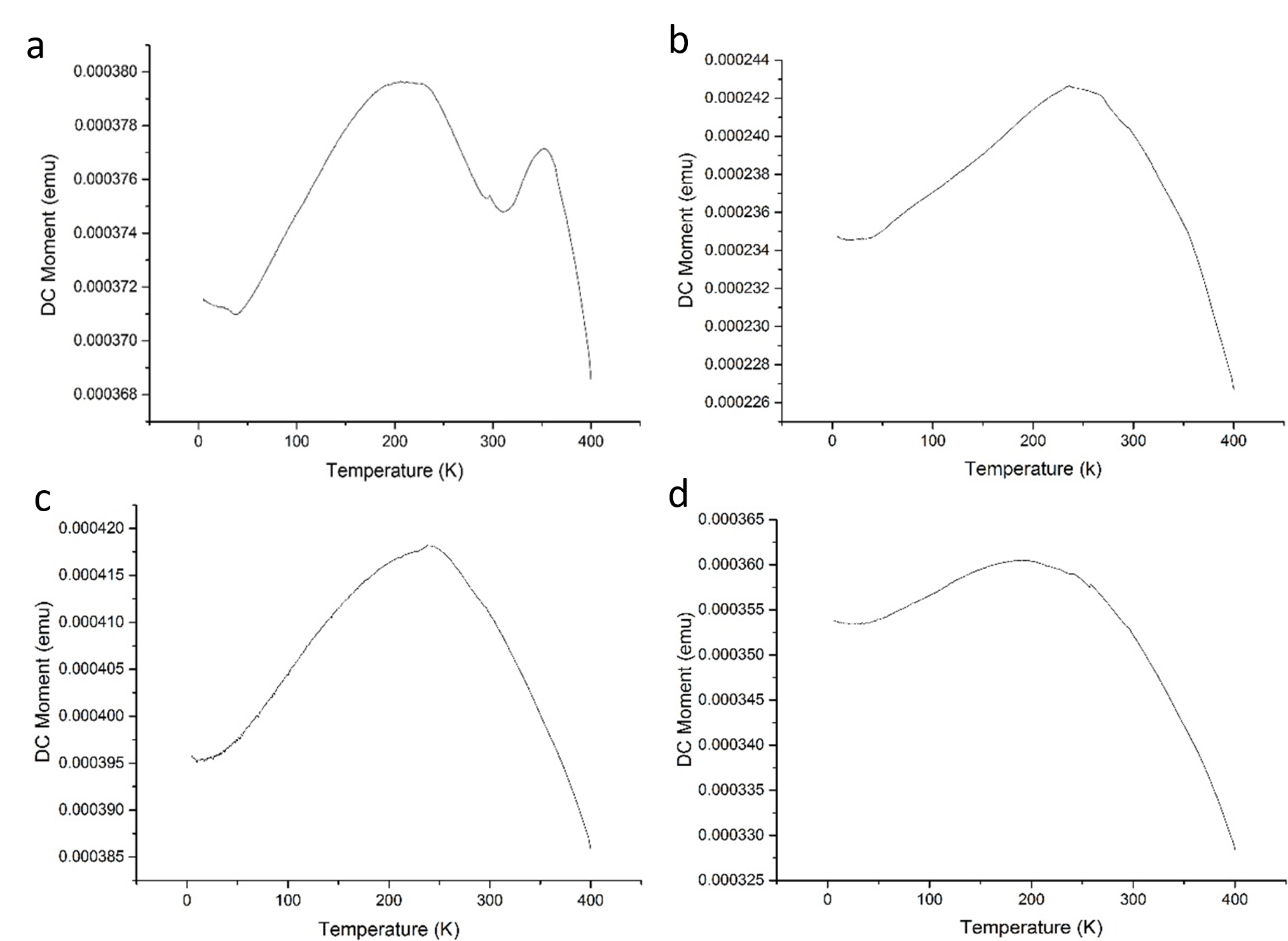

Supplement: S4 Fig — Magnetization measurement of SPIONs (FC mode) through the coreshell preparation: (a) naked SPIONs, (b) after functionalizing with SDS, (c) after curcumin loading, (d) after encapsulating with biopolymer coating. (TIF) [file pone.0200440.s004.tif]

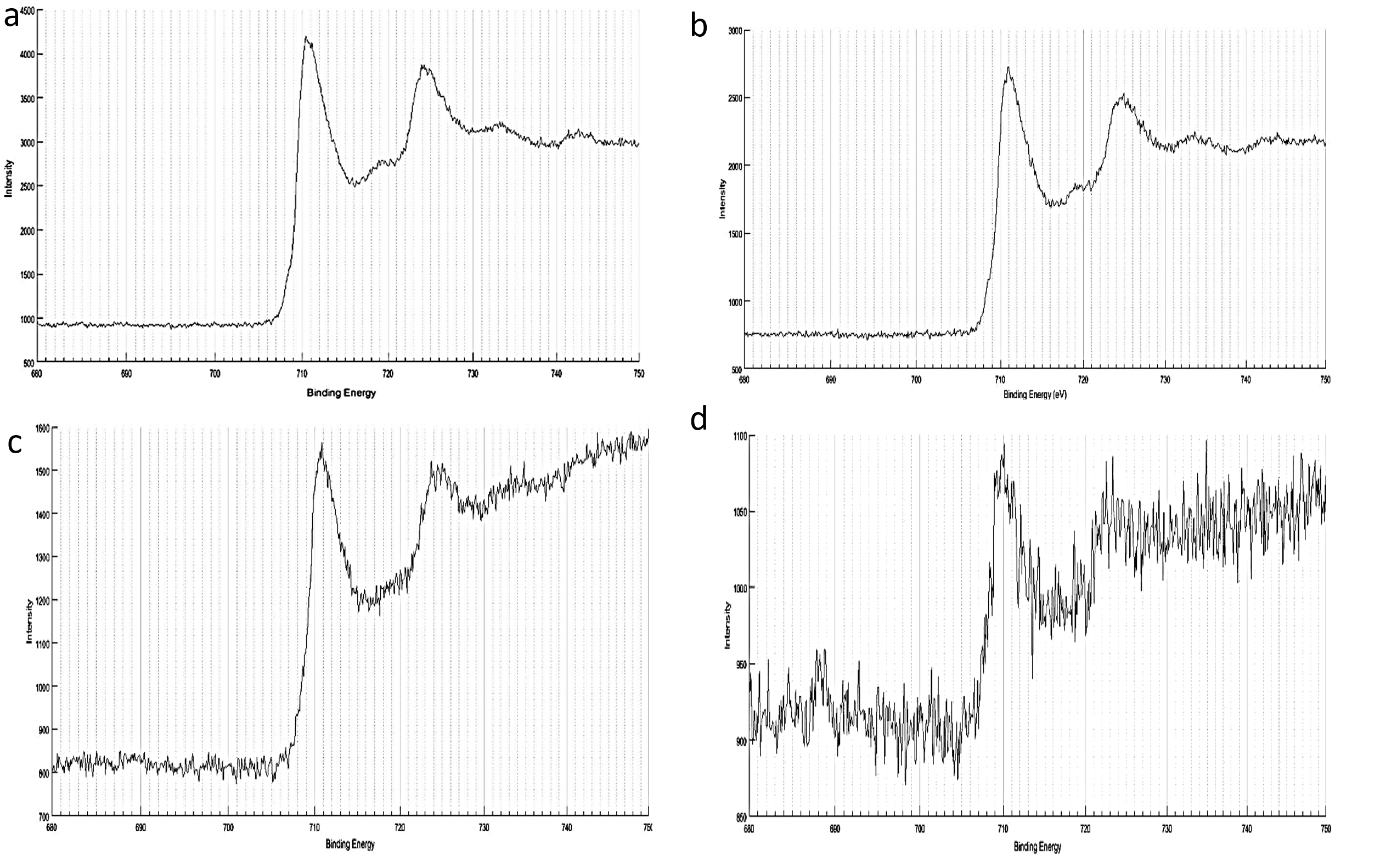

Supplement: S5 Fig — XPS spectra for the chemical states of Fe over the phases of coreshell preparation: (a) SPIONs, (b) after functionalizing with SDS, (c) after loading curcumin, (d) after encapsulating with biopolymer coating. (TIF) [file pone.0200440.s005.tif]

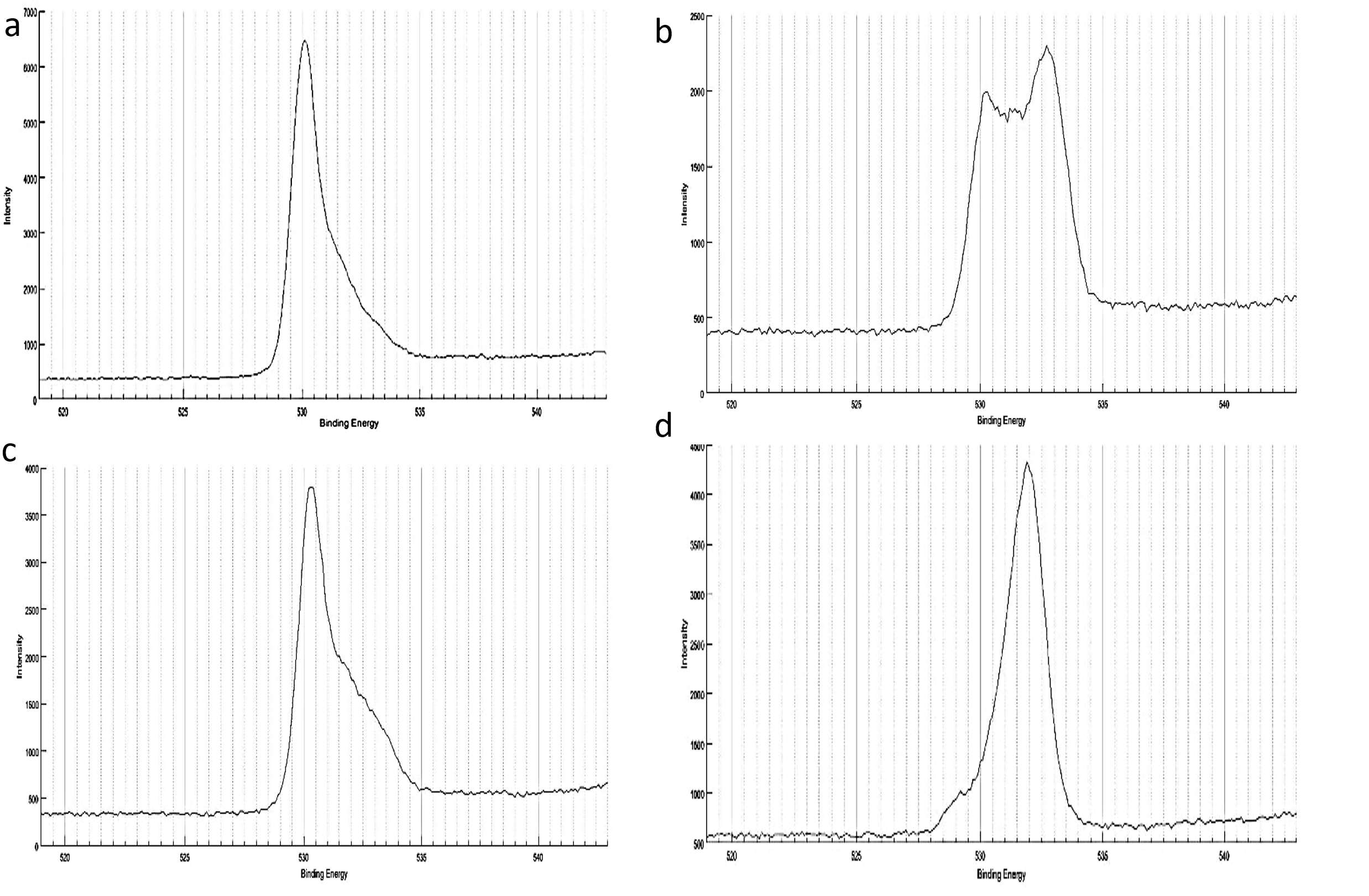

Supplement: S6 Fig — XPS spectra for the chemical state of oxygen over the coreshell preparation: (a) SPIONs, (b) after functionalizing with SDS, (c) after loading curcumin, (d) after encapsulating with biopolymer coating. (TIF) [file pone.0200440.s006.tif]

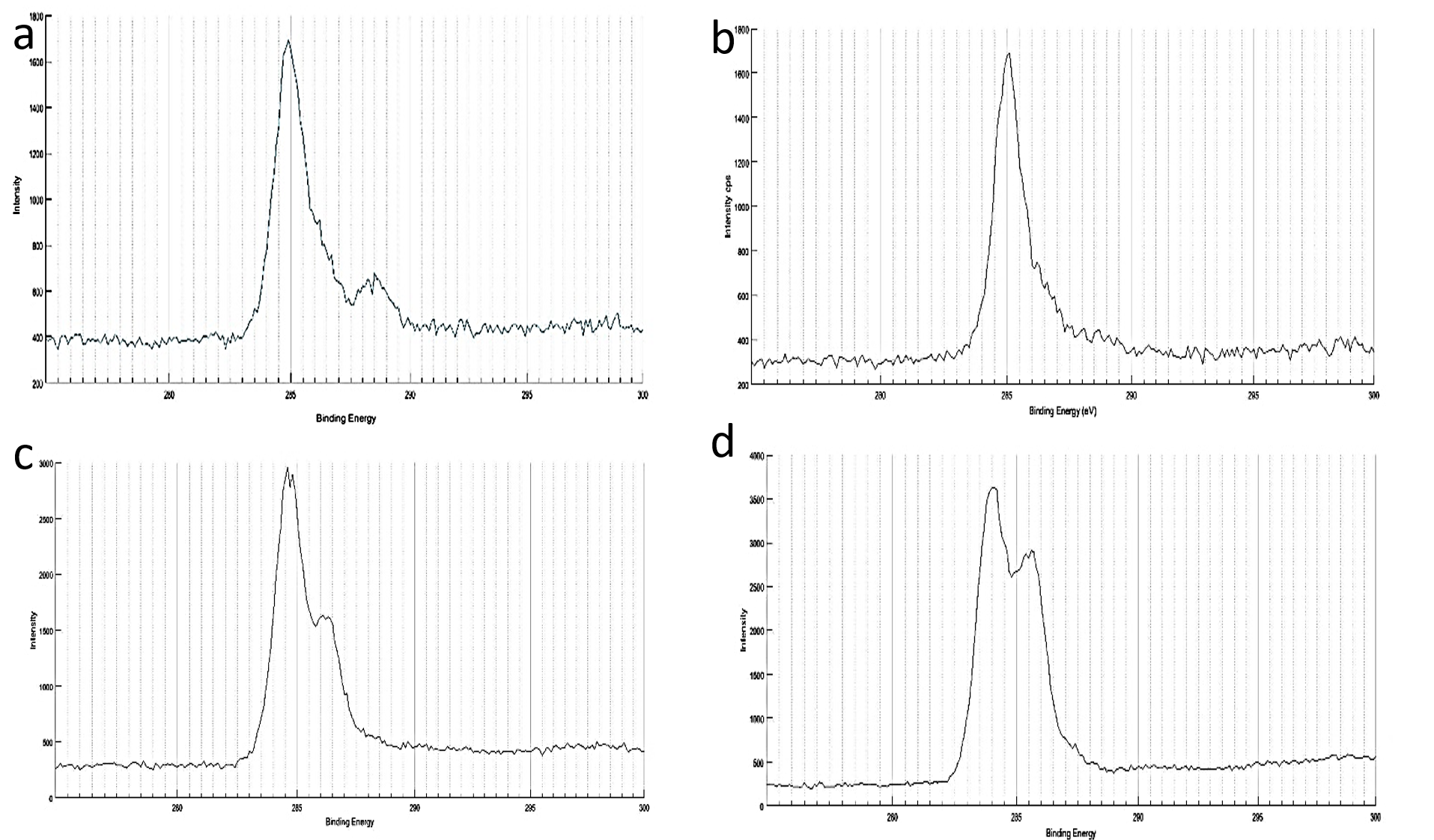

Supplement: S7 Fig — XPS spectra for the chemical state of carbon over the coreshell preparation: (a) SPIONs, (b) after functionalizing with SDS, (c) after loading curcumin, (d) after encapsulating with biopolymer coating. (TIF) [file pone.0200440.s007.tif]
